# The Impact of Modern Ultramarathons on Shaping the Social Identity of Runners. The Case Study of Karkonosze Winter Ultramarathon

**DOI:** 10.3390/ijerph17010116

**Published:** 2019-12-23

**Authors:** Marek Kazimierczak, Agata Dąbrowska, Katarzyna Adamczewska, Ewa Malchrowicz-Mośko

**Affiliations:** 1 Faculty of Sport Sciences, Poznan University of Physical Education, Krolowej Jadwigi 27/39, 61-871 Poznan, Poland; makazim@o2.pl (M.K.);; 2 Faculty of Health Sciences, Poznan University of Physical Education, Krolowej Jadwigi 27/39, 61-871 Poznan, Poland; adamczewska@awf.poznan.pl

**Keywords:** sports events, ultramarathon, social identity, sports subculture, authenticity of experience, sports motivations

## Abstract

Despite the growing interest in extreme sports around the world, researchers have rarely investigated the complex factors that have led to a developed commitment to extreme sports in recent years. Precisely, the social identity of ultramarathoners remains a research niche. The aim of the article is to analyze the impact of a sports event on shaping social identity of ultramarathon runners on the example of Karkonosze Winter Ultramarathon (held in Poland). The qualitative method used in the article—interviews with runners—made it possible to examine the factors that create social identity, among which the motives for participation, sports subculture, and the authenticity of the experience play a key role. The first part of the article describes the theoretical aspects of social identity in sport. The second, empirical part presents the research results supplemented by the statements of the contestants. In this case, the subject of analysis is the motives for participation in a winter ultramarathon and their characteristics. Lastly, the article analyzes the subculture of ultramarathoners and the experience of contestants’ authenticity. The investigated winter ultramarathon created the perfect space for creation, deepening and celebrating the social identity of ultramarathoners assessed as a value in itself. The article enriches the present knowledge about the motivation of ultramarathoners because, unlike the results of quantitative research, it presents in-depth responses of runners who were not always concerned by existing research questionnaires.

## 1. Introduction

Sports development is being increasingly embraced as part of a broader philosophy of sustainable development which focuses on improving quality of life, tackling social exclusion, increasing access, preserving the environment, and expanding the pursuit of excellence—running can serve as an example. According to Zhou et al. while interest in extreme sports is rising, few studies have investigated the motivations of athletes in extreme sports. Moreover, reasons leading to develop extreme sports involvement over the years are very complex [1]. In particular, the social identity of ultramarathoners remains a research niche. Many amateur athletes who travel to participate in events can be described as active sports tourists and this has led to the notion of serious sports tourism [2]. This line of theory development relies mainly on serious leisure as developed by Stebbins [3]. A sports event, by providing authentic, extraordinary experiences, creates an opportunity for sociocultural reflection on the elements that create social identity of its participants. 

The aim of this article is to determine the social identity of ultramarathoners based on the experience of participants to Karkonosze Winter Ultramarathon. In today’s society, ultramarathoners have the image of strong, well-trained, and achievement-oriented people striving for a goal [4]. The analysis of the factors that create the social identity is accompanied by the following research questions: How do the individual motives for participating in a sports event influence the process of creating social identity among contestants? How do ultramarathoners characterize their own subculture? What significance does the event experience have during the extreme psychophysical effort for the participants of the event?

In our work, the analysis of the factors that create social identity has been referred to as one of the most significant social phenomena of our time, i.e., long-distance running. The authors of the present article consider the research goal to be important from the point of view of social sciences, namely as sporting events are special events that reflect individual lifestyles by means of extraordinary experiences of a collective nature, providing them with the possibility to stabilize their own identity. As events that are a reservoir of principles, rules, and values, they express a self-selected and aesthetically organized lifestyle. At the same time, they offer the opportunity to stabilize and shape their own identity in the face of existing practices of living a specific lifestyle. Through their atmosphere and mood, they express a sense of being together with other participants in the event. Identity can be also developed as a long-distance runner without participating in sports events.

Among the factors that create social identity, the motives for participation, subcultures, and the authenticity of the experience play a key role [5]. For its participants, a sports event is an emotional experience (providing authentic sensations) that combines a unique atmosphere with a specific subculture. Sports activity plays a significant role in the construction of social identity. The characteristics of serious leisure activities include high self-esteem, self-confidence, self-respect, self-realization, creation of an ethos that defines norms, values, and attitudes of great value for the ultramarathon subculture that distinguishes them from the rest of society [4].

Representatives of classical identity theories [6] distinguish personal and social identity. They argue that both personal identity and social identity are developed and is the result of social interactions and expected roles. Social identity refers to super personal characteristics of a human as a carrier of specific roles, an owner of a specific status, or a member of a particular cultural group. It is an expression of social belonging and is understood as collective self. Group identity consists of defining yourself by belonging to various types of social groups. Individuals construct themselves in the area defined by the culture of a given group and community. It is the group or community that provides specific categories to describe oneself [7].

Tajfel’s social identity theory says that people strive to maintain a positive self-esteem. This means that self-esteem is achieved partly by positive self-differentiation from others—this is the so-called need for self-enhancement of the individual. Moreover, the concept of self mainly results from the identification with the group, while a positive social identity arises as a result of intergroup comparisons [8].

According to Turner’s self-categorization theory, personal identity results from specific, individual properties and interpersonal relationships with other individuals. The self-category is seen as a unique individual in terms of differences between them and members of their own group. On the other hand, social identity understands the category of self as an individual that is similar to other members of their own group and at the same time different from members of outside groups. Group goals, values, and norms are their common features [9]. Activation of identity depends on various factors, including mainly the situation and context (e.g., interaction with members of other groups).

Belonging to a given group and social category often means having many social identities. Traditionally, social identity is identified by assigning the individual to the category of family, religion, or work [10]. Social identity of a participant in a particular subculture is demonstrated through language, clothing, norms, and values [11]. Furthermore, it presents an individual as a member of the group and describes specific behaviors.

Social identity refers to the supra-individual record of people with a certain status, members of a particular cultural group [2]. It is an expression of social belonging and is identified as “collective self.” There are many reasons why social identity is of great value to an individual. In addition to providing it with a sense of belonging, an important place in the social environment and the ability to connect with other people, it strengthens self-awareness and self-esteem in the individual [7].

Beyer defines the particular importance of identity and sports in three points. First, sports offer the possibility of creating and confirming different forms of identity. They depend on the “quasi-experimentally” constructed sports conditions in which the individual sports experience takes place. Secondly, sport provides individuals with opportunities to demonstrate and make significant their advantages, and in a way that does not cause changes that may lead to threats in the area of social existence and power relations. Thirdly, sport gives various social groups a basis for creating. A collective identity expressed in many ways [12]. Through sport, it appears possible to experience simultaneously ambivalent phenomena, such as integration and exclusion. One possible explanation for the social need to identify with a group in sport and through sport is the social desire for spontaneous, thoughtless worlds of sensation in controlled civilized societies [13]. Sport provides numerous opportunities for this.

**Scheme 1:**
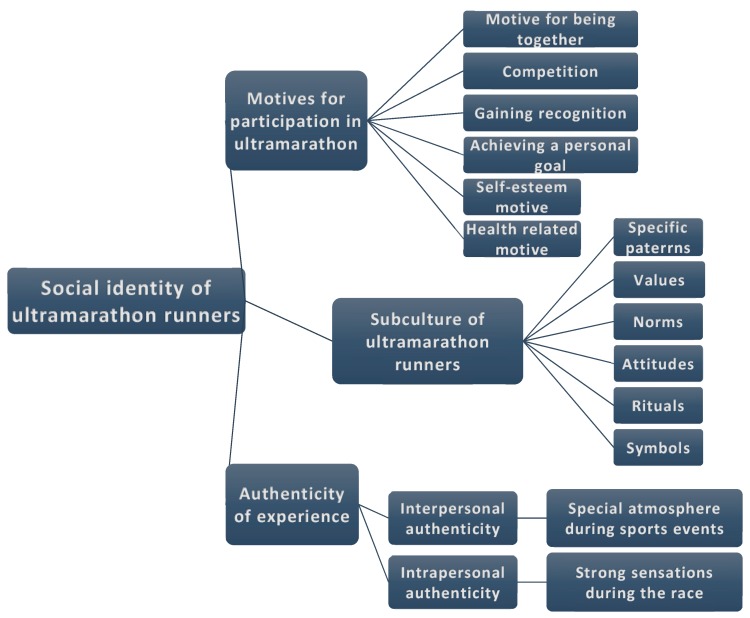
Social identity of ultramarathon runners—own elaboration.

Subculture exerts a significant influence on its members through specific values with which attitudes and norms are associated [14]. In the area of sports activity, symbols that show how a player identifies with a particular sport and thus express his identity in a specific way play an important role. For example, participation in a running event can symbolize a sports lifestyle, discipline, and motivate sports activity. Perseverance, the ability to achieve goals, independence, self-discipline, and endurance are very important as values in the runners’ subculture [15,16]. Usually, the status of a runner in the runners’ subculture is defined by the best times obtained, the number of runs completed and the prestige of the place where they are held [7,15,16]. These factors make up a kind of *subcultural capital* of a runner [17]. Below the results of our study on attitudes of ultramarathoners towards sports subculture, the authenticity of the experience, and motives for participation in ultramarathons are presented. The scheme of social identity of ultramarathon runners has been presenten in the Scheme 1.

## 2. Research Methodology and Characteristics of the Research Sample

The Karkonosze Winter Ultramarathon due to the season in which it is organized (March, winter time in Poland) is not an ordinary long-distance run. The route of Karkonosze Winter Ultramarathon runs through the entire Karkonosze Mountains ridge and is 52 km long. The first edition of the event took place in 2014. The run attracts 200–300 runners every year. Due to the possibility of experiencing true winter weather, the time limit for covering the entire circuit is 11 h. Highly varied terrain, large differences in elevations, and the wintertime conditions make this race one of the most difficult and demanding contests. Four nutrition points were organized, spaced approximately every 10–15 km. Stops between these points were not advised—even a short break on the route without shelter may be associated with rapid cooling down and loss of will to run. Participants in the competition have to have experience in long-distance mountain races (they know their body, skills, and are prepared to compete in difficult conditions) and also know what is associated with moving in the winter in the mountains. Low temperature, snowfall, wind, fog, long-term struggle with psychophysical discomfort are just some of the challenges faced by contestants on the ultramarathon route. The goal of the ultramarathons’ organizers is to create extreme races that question the common concept of endurance of the human body. The information about equipment and safety is presented in Appendix A.

The Karkonosze Winter Ultramarathon is dedicated to the memory of a friend of the organizers—Tomek Kowalski, who did not return from the Winter Expedition to the 12 highest mountain on Earth—Broad Peak on March 2013. Kowalski climbed six of the nine “Seven Summits” mountains. He made each of his climbs unique and something more than a simple ascent. In 2009 he successfully climbed the three highest six-thousanders of South America during a solo adventure. In 2010 traversed the massif of Mount McKinley—the highest mountain peak in North America—and in 2011 he solo-summited the four highest mountain peaks of the former Soviet Union—the so-called “Snow Leopard”—within a record period of 28 days. He completed mountain ultramarathons and adventure races all over the world. He also loved to travel. In 2010 he took an 18-month trip around the world visiting several dozen countries. Kowalski was a member of a 2013 winter expedition to Broad Peak. Polish mountaineers attempted to make the first historic ascent of Broad Peak in harsh winter conditions for two months. On 6 March, after reaching the summit of Broad Peak, Tomek Kowalski and Maciej Berbeka went missing on the decent and never made it back to the camp. They remain in the mountains forever—on 15 July, Jacek Berbeka and Jacek Jawień found Tomek’s body. They buried him on the mountain crest, below the Rocky Summit. Today many young runners—Tomek’s friends—take part in the Karkonosze Winter Ultramarathon.

The qualitative content analysis was used during the research. It is a technique applied to examine text and oral messages. Data analysis was carried out with the help of a theoretically developed system of categories in which relevant statements and fragments are arranged. The categories were harmonized, reviewed, and changed, if necessary, with the data. The strength of qualitative content analysis lies in the gradual and methodically controlled material analysis.

The qualitative analysis carried out includes 12 interviews with participants of ultramarathons. When selecting people for the interview from 242 runners, the following criteria were met: the participant had to be willing to participate in the interview and had experience in participating in at least one run of such type. A partially structured interview according to specific thematic blocks was prepared for participants. The order of questions, as well as the time devoted to each thematic block, was adapted to the individual situation of the respondent. The researcher properly directed the conversation in which open questions allowed freedom of expression. The interview time was about 20 min of conversation recorded on a Dictaphone. The study was conducted after the race in Karpacz (the meeting place of participants). All the questions are presented in Appendix A.

The description of the research sample together with the characteristics of contestants are presented in Table 1.

## 3. Empirical Research Results

Below we present and characterize the components of social identity, i.e., motives for participation, subcultures, and the authenticity of the experience based on research data collected during the ultramarathon.

### 3.1. Motives for Participating in Ultramarathons Related to the Process of Creating Social Identity

Over the last few years, an increasing number of sports and recreation running events designed for amateurs was observed. This raises the question of what drives people who choose to participate in these sports activities. Many quantitative research methods have been created to describe the motives for participating in marathons (e.g., MOMS—Motivations of Marathoners Scale) [18]. In our article, the authors refer to a study by Stoll, where selected motives for participation are associated with shaping and strengthening social identity. Stoll divided the motives for participating in the sports event into (1) motive for being together; (2) motives for gaining recognition; (3) competition motive; (4) motives for achieving a personal goal; (5) self-esteem motive; and (6) motives for improving health [19]. Participants answering the questions related to the mentioned motive for participating revealed many appreciable behavior and attitude characteristics of the ultramarathoners’ subculture and their identity.

The first of the motives highlighted by the authors of the mentioned paper is the motive for being together. People fulfil themselves socially by participating in sports activities with friends and competitors similar to them. Social interactions, being together with friends, and belonging to a group of ultramarathoners becomes a premise to develop a socially valuable identity. Communication with other runners during competitions that share a common interest initiates the establishment of many lasting relationships, which is confirmed by the following statements of the respondents: “most of the people I keep in touch with are people I met thanks to my passion for running” (Tomasz), “We meet someone new at every competition, many of these friendships have survived to this day” (Katarzyna [2]). Being together with similar people as well as belonging to an ultramarathon group helps strengthen social identity. 

Another motive is recognition, which is one of the basic human needs. An ultramarathon allows event participants to present themselves from the best side, as a result of which they receive a social reward in the form of recognition of other runners, fans, volunteers, friends, family, journalists. The following statements testify to this: “We are always happy when someone looks at our struggles and encourages us” (Mateusz), “fans are very important on the route! They bring energy, motivate to continue running. I have often found out on the route how cheering can put a man back on his feet” (Tomasz). When asking contestants about their social perception among their immediate family and friends in the context of recognition for their accomplishments, in most cases positive answers were obtained, among others: “people sometimes ask with disbelief how it is possible to finish such. A marathon, run such a distance in the mountains… At the same time, they admire and motivate us to further training” (Wojciech). The award of universal recognition leads to the fact that social identity of the constant is increased, and their accomplishments are admired and publicly valued. In today’s society, ultramarathoners have the image of strong, well-trained, and achievement-oriented people striving for the goal. 

The competition motive, i.e., the possibility of competing against other ultramarathoners, is for the participants of the sports event an important reason why they are intensively preparing the whole season. In the runner community, professional runners strive to compete with other competitors or run only for themselves. For one of the contestants’ competition means “the desire to be faster than the opponents (…) and satisfaction from defeating a person who was better so far” (Agnieszka). Event participants emphasized that competition is an opportunity to test yourself and compare with other contestants. The ultramarathoners often said that they “compete against oneself. They don’t run in ultramarathons to win high places or prizes. It is rather a desire to overcome one’s own weaknesses” (Piotr [2]). Overcoming one’s own psychophysical barriers is the core of the matter among the participants of ultramarathon: “competition with someone is important, but even more important is competing with oneself. The factor of self-improvement is very important for each of us” (Katarzyna [1]). The more success the participants achieve in a sports competition, the more positively their social identity is assessed by other runners and fans. 

The motive for achieving a personal goal is for the most part an internal source of inspiration for action. Motivated and determined contestants are extremely worthy for their social identity as a group. Satisfaction with achieving the desired goal as well as critical self-assessment when achieving an unsatisfactory result is a strong incentive for the contestant to increase effort during subsequent preparations for ultramarathon races. This is confirmed by the words: “testing myself and my own skills is for me my verification—whether I’m going ahead or standing still. I can prove how much I can achieve and whether I have used the time for training and preparation well” (Agnieszka). Facing a new, unusual challenge is also a detachment from ordinary everyday life, about which the participant of the ultramarathon recalls: “For me, achieving a personal goal is the meaning of life (…) something that distinguishes our daily activities” (Mateusz). The implementation of set goals is very important for the development of contestants’ social identity, as it reflects the continuous improvement of psychophysical abilities of its participants. 

Another motive is self-esteem, which expresses how individuals assess themselves, with higher self-esteem resulting in a more positive self-esteem. By practicing long-distance running, the contestant’s attitude to their own body and skill changes, which is demonstrated by the words: “in periods of increased training activity, self-esteem increases significantly. A person feels invincible, even euphoric” (Mateusz). The most effective way to develop a strong belief in one’s own effectiveness is to experience fulfilment—to achieve the intended goal, which increases self-esteem, while the awareness of non-fulfilment causes doubts about self-worth. 

Another important motive is health with its physical and mental dimension, which is particularly strongly marked in the subculture of runners, whose identity implies specific behaviors in this sphere. A healthy, trained, and strong body is in our society a synonym of an ideal to which every athlete aspires. Interviewed runners reported that since they started running, they have been feeling physically and mentally well and rarely have gotten sick: “regular training helps maintain good condition, physical health and increases immunity. It also has a cleansing effect on the psyche. Regular training provides contestants with vitality, teaches regularity, responsibility, relieves feelings of stagnation and ineptitude” (Tomasz).

### 3.2. The Subculture of Ultrarunners in the Process of Creating Social Identity

Sports events are characterized by emotional experiences that create a unique subculture through a special atmosphere, episodic nature, participation, and a sense of community. Subculture, understood as a community whose members identify with each other, defines specific patterns, values, norms, attitudes, rituals, and symbols. When asking ultramarathoners questions about their community, they most often referred to shared experience, mutual communication, and encouragement. An open, friendly, and full of solidarity atmosphere during the sports event is conducive to building lasting social relationships, which is confirmed by the words: there is a family-friendly atmosphere among the contestants. Numerous examples from the route prove this: “when you run fast and pass someone who runs very slowly, you can always hear words like—friend, brother, are you all right? Do you want to drink, do you want to eat, do you need something? …” (Piotr [1]). Participants are aware of many hazards on the race route, therefore everyone is trying to consider the other person, which is proved by the statement: “On the road I feel very badly—a contestant with whom I raced for a long time, despite this unfortunate event, waited for me and asked if everything was all right and whether I need his help” (Krzysztof). Belonging to the ultrarunner subculture implies specific behaviors, attitudes, and values as demonstrated by competitors during extreme struggles. When asking participants what they talk about in their free time during the event and how these conversations influence the subculture, the following answer was received: “we are talking about shared experiences. By looking at photos, watching films from the competition we return to great moments. It is something that will stay with us for the rest of our lives. No material thing can replace such experience for us” (Piotr [2]). Hence, naturally, the question arises about the importance of awards in the lives of ultramarathoners. For some, medals, cups, diplomas, and in-kind prizes are very important, whereas for others they do not matter: “you don’t come here for prizes. It is important to participate, finish the run, meet friends” (Hanna), “I never paid attention to material prizes. They are certainly a lot of fun, but they are not a priority (…)” (Tomasz). Completing the ultramarathon is for many competitors the culmination of their many months of titanic work. This kind of achievement says a lot about a person and their character as evidenced by the following statements: “it is somewhat a testimony of a man who pursues a goal. It will not always be possible to achieve it, but trying itself already shows a person’s character, strength, discipline and self-determination” (Wojciech), “not so much the completion of the ultramarathon as the entire preparatory process. This is not one run, it is a whole series of training that you must absolutely do to be prepared and think about finishing the competition (…)” (Radosław). In the contestants’ lives running plays a very important role in relation to other spheres such as work, family, religion, or politics: “running is part of our identity. You strongly identify with it (…) This is something without which life would lack its savor” (Mateusz). When creating the psychophysical profile of the ultramarathoner, the contestants listed the following features: steadfast, determined, resolute, confident, stubborn, disciplined, resilient, brave, valiant, resistant to pain and their own weaknesses—all these features are extremely highly valued in the sports subculture.

### 3.3. The Authenticity of the Experience during a Sports Event in the Process of Building Social Identity

A sports event like an ultramarathon is a very emotional experience that provides many extremely authentic experiences and indicates the sense of overcoming own psychophysical barriers. These types of experiences undoubtedly build a sense of self-worth of participants, making them stronger and more resistant to the hardships of everyday life, which confirms the statement: “the authenticity of experience and the sense of overcoming my own weaknesses are very important to me. I shape my character and face my weaknesses. The stronger satisfaction I feel when running into the finish line undefeated” (Piotr [2]). By pushing the boundaries of the psychophysical capacity of the runner further and further, an ultramarathon causes that the appreciation of the body and the deepening of experiences with one’s own body result in strengthening the identity. The unique atmosphere of the event, conditioned by spectators and observed by contestants’ dramatic tensions, reflects the celebration of the social identity of ultramarathoners, a fact mentioned by Piotr: “(…) I feel very well with the awareness of being an ultramarathoner. A person feels certain uniqueness. During a running event a person does not only find authentic experiences with oneself (intrapersonal authenticity), but also looks for authentic time spent together with others (interpersonal authenticity) (…)” (Piotr [2]). The opportunity to experience one’s runner’s identity and gain recognition from spectators increases the participants’ satisfaction with the sports event and becomes the guarantee of the success of its organizers. Experiences and sensations during ultramarathon lead to the fact that the participant of the event creates a fully recognized social identity. When asking contestants whether the genuine experience of participating in ultramarathon affects the social identity of ultramarathoners, most responded that it definitely does. Authentic positive experience is certainly provided by the fact that running takes place in a natural setting, close to nature and among people with a similar lifestyle [20,21].

## 4. Ultramarathons—From Extreme to Mainstream: Discussion

Mass sports events are now extremely popular among many sports enthusiasts. Currently the most popular mass sports events include among others marathon runs, cycling events and physically demanding triathlons and ultramarathons. The dynamic development of mass sports events comes with questions about the motivations of the postmodern man to participate in them. Recently, researchers focused mainly on mass, popular, street runs/cycling events in the context of their meaning for sporting events and active tourism or in the context of motivation for running/cycling or health implications for runners/cyclists [22,23,24,25,26,27,28,29]. Sporting events have been analyzed as a tourist phenomenon [30,31,32,33,34] and the social identity of athletes is rarely investigated in this area. The dynamic growth of running sports events is a result of different factors. We may highlight the search for strong, authentic emotions and experiences, among others, which are brought to us through sporting rivalry and the possibility of making social relationships (the need to feel unity during such events) in a period of time where we live in a society tinted with individuality. People taking up sports and recreational activity more frequently feel the need to increase their training loads and enhance their efficiency, participate in situations posing an opportunity to verify their own accomplishments, constantly check back, and achieve a high level of stimulation. Such a phenomenon can nowadays be one of the most important factors influencing the choice and effectiveness of various forms of sports and recreational activity. According to the outlined direction of developmental changes in contemporary sports and recreational activity—from recreation to excitement—participants are increasingly looking for excitement related to self-improvement, competition, and even physical pain, and, above all, experiencing exciting and intentionally controlled risk. Ultramarathons and triathlon events create an opportunity for maximum physical and mental effort for representatives of the generation of pain. Hanson et al. [35] underline that ultramarathoners more often than contestants of marathons and half-marathons indicate psychological and social motives than these connected with physical health orientation and weight concern. The Karkonosze Winter Ultramarathon due to the season in which it is organized (winter) is not an ordinary long-distance run. Low temperature, snowfall, wind, fog, long-term struggle with psychophysical discomfort are just some of the challenges faced by contestants on the ultramarathon route.

Rohm et al. classified several types of runners [36]. Serious leisure runners were attracted by mental and physical-related factors; however, social motives differentiated them from the other athletes. Investigated ultramarathon runners claim that regular training has a positive effect on physical and mental health and has a cleansing effect on the psyche. There is also some evidence from previous studies that running has a positive impact on mental health and causes feeling of catharsis [1,26]. Moreover, the satisfaction with one’s running progress mediated relationships between well-being and the amount of running. Increase in training led to increase in satisfaction with own progress, which leads to increased personal happiness and well-being [37,38]. This could explain the addiction of ultra-marathoners to running. According to Waśkiewicz et al. ultramarathoners had higher orientation on affiliation and life meaning than on weight concerns, personal goal achievement, and self-esteem [39]. Ferrer et al. discovered that older runners are more motivated to training for the physical factors than younger ultramarathoners [40]. According to Hoffman and Krouse [41] the high percentage of ultramarathoners would not stop training even if they learned it was not good for their physical health as it appears to serve their personal achievement and psychological motivations and their challenge orientation such that they perceive enhanced positive effects that are worth retaining at the risk of their health condition. The rivalry factor, although it has always been more important for men, has recently gained in importance also among women [42]. The article enriches the current knowledge about the motivation of ultramarathoners because, unlike the results of quantitative research, it presents in-depth responses of runners who were not always concerned by existing research questionnaires (e.g., MOMS scale). These motivations constitute social identity of runners, together with a subculture of runners and authenticity of experience. Respondents’ statements confirm the thesis that social identity is of great importance for ultra-runners and they undertake the hardships of training not only because of physical health.

One of the key premises of social diagnoses describing life in the experience society (Erlebnisgesellschaft) is to notice a shift in social processes towards individualization, which is accompanied by an increase in the flexibility of behavior in the sphere of free time. Cultural patterns and aesthetic forms of expression that combine events into a unified whole assume a performative and expressive-symbolic character. “Being present, participating in something special,” ‘seeing and being seen’ becomes the dominant behavior [43]. Emerging sports performances, which are forms of dramatic presentation of not only sporting experiences, contribute to the projection of alternative social ways of behavior. As collective, temporal projects (compositions), sporting events are used to socialize identity, mediating the formation of a sense of community, a kind of *communitas*, releasing various feelings and moods [44].

Participation in the ultramarathon run, which is a kind of “ultramarathon feast,” creates an opportunity to meet the personal identities of the runners’ subculture. On the marathon “stage” of psychophysical experiences, their authenticity is confirmed, which leaves traces in the world of runners often going beyond this “stage.” The category of “authenticity” plays a key role in describing such a specific sphere of sporting experiences generated by the ultramarathon run. The state of experiencing authenticity is a state of remaining “true to oneself” [45]. The ultramarathon run, pushing the boundaries of the runner’s psychophysical capacity more and more, means that the appreciation of the body and the deepening of experiences with one’s own body result in strengthening the identity [15,16].

The world of ultramarathoners is not available to everyone, because being in it is associated with demonstrating uncommon loyalty to the chosen sport discipline, which requires strong will and fortitude. Staying in such a specific sports microworld, in return, offers runners a deepening of personal identity, enabling authentic rooting in the community of other running enthusiasts, and on the other hand, broad social recognition, especially for those who do not lose hope that they may someday join them. Previous studies show that a running event has the potential to encourage for running people who have not been physically active before the event (e.g., inactive supporters) [46].

## 5. Summary and Conclusions

Creation of social identity comprises a number of factors. The research results were grouped into three categories: motives for participation, subcultures, and authenticity of the experience. A sports event creates the perfect space for creation, deepening, and celebrating social identity of contestants assessed as a value in itself. In the ultramarathoner subculture, a strong need for integration with contestants, who could share their feelings and experiences along with others, was identified during the event. These are the factors that build the so-called social basis of identity, thus providing extraordinary and authentic experiences. However, the world of ultramarathoners is not available to everyone, as being a part of it requires demonstrating uncommon loyalty to the chosen sport discipline, which requires strong will and fortitude. Researchers also indicate that participation in running reflects on individualization and post-materialism processes in society [47]. Running events become a postmodern form of participation in social life—they allow to feel part of the community of runners. The need for affiliation is now extremely important in Western societies, which currently do not create many possibilities for collectivist behavior, as in Eastern societies. In the face of the weakening of traditional social environments in Western cultures and the progressive individualization of society, the individual, not wanting to be self-reliant, actively seeks new social structures for himself. People are looking for contact with other people who lead a similar lifestyle with similar interests and similar views. Their physical presence, the ability to visually and tangibly ensure their existence, is at the same time making sure that an individually chosen lifestyle works. Social occasions for such meetings are created by sports events. Taking part in a sporting event usually involves a strong sense of community with other participants, it allows sharing emotions with people around. In the modern world, in which we observe the aspiration to atomize society, full of loneliness and loss of postmodern people, and full of problems with establishing and maintaining interpersonal relationships, sporting events create opportunities for building a social relationship and strong social identity. Sports events as meeting areas are important. Sports events that are ostensibly devoted to rivalry also provides possibilities to interact with other athletes sharing the same bundle of motives. Some events can be closely associated with social worlds because of their concentration of actors, practices, or events, implying that a destination could become a Mecca for runners or for those pursuing challenging outdoor pursuits in general.

A sporting event creates space for building and deepening social identity assessed as a value in itself. In the case of active participation in sports activities, the athlete’s *habitus*, his cultural and social capital play an important role. The characteristics of serious leisure sports activities, which are well expressed in the concept of *serious leisure*, include perseverance in the pursuit of self-realization and sports fulfillment, building a peculiar ethos that is so significant for members of the ultramarathoners’ subculture.

## Figures and Tables

**Table 1:** Personal data of ultramarathon contestants.

Interviews with Contestants of Karkonosze Winter Ultramarathons (KWU)												
Contestant Personal Data	Interview No. 1	Interview No. 2	Interview No. 3	Interview No. 4	Interview No. 5	Interview No. 6	Interview No. 7	Interview No. 8	Interview No. 9	Interview No. 10	Interview No. 11	Interview No. 12
Name of the contestant	Hanna	Krzysztof	Piotr [1]	Tomasz	Radosław	Piotr [2]	Katarzyna [1]	Karolina	Mateusz	Katarzyna [2]	Agnieszka	Wojciech
Age	61	34	28	27	41	35	26	32	32	30	28	30
Sex (F/M)	F	M	M	M	M	M	F	F	M	F	F	M
Education	Higher	Higher	Higher	Secondary	Higher	Higher	Higher	Higher	Higher	Higher	Higher	Higher
Occupational status	Professionally active—own company	Professionally active—own company	Unemployed	Professionally active	Professionally active	Professionally active	Professionally active	Professionally active	Professionally active	Professionally active	Professionally active	Professionally active
Years of training long-distance running	2	3	3	3	3	2	4	1	3	5	4	4
Frequency of training (number of times a week)	2×	4–5×	4–5×	10–12×	4×	Min. 3×	6×	6×	4–6×	4×	4×	3×
Number of times they have participated in KWU including this one	2	2	2	1	1	1	1	1	1	1	2	1
How they assess their preparation for ultramarathon	On average	On average	On average	Good	Very good	Good	Very good	Very good	Very good	Good	Good	Good
How often they participate in similar ultramarathons (number of times a year)	4–5×	6×	4–5×	2–3×	2–3×	1–2×	A few times	3×	2×	2×	5× only in the summer season	3×
Are they planning to take part in KWU next year	Yes	Yes	Yes	Yes	Yes	Do not know	Do not know	Rather not	Yes	Yes	Yes	Yes

## Appendix A

1. What motivated you to take part in the Karkonosze Winter Ultramarathon?
2. Is ultramarathon an opportunity for you to spend time with your friends? What does this mean to you?
3. What does the presence of supporters on the route and at the finish line of the ultramarathon mean to you?
4. How do your friends perceive your participation in ultramarathon? Do you enjoy their recognition?
5. What does rivalry with others mean to you?
6. What is the meaning of competition with yourself and the opportunity to test yourself and your own skills?
7. What does achieving the goal mean to you?
8. Does the opportunity to overcome your own weaknesses by participating in Karkonosze Winter Ultramarathon build self-esteem?
9. How does long-distance running affect your health?
10. Do you think participation in extreme running events is currently fashionable?
11. What is the atmosphere like among runners during the ultramarathon?
12. Did you meet new people? What does it matter?
13. Do your ultra-marathon friends participate in the competition?
14. What do you talk about in your free time? Can you find in these conversations the values characteristic of the community of ultramarathoners?
15. What significance do the prizes that you can get in ZUK have for you?
16. What do you think completing an ultramarathon says about a runner?
17. How would you characterize an ultramarathoner? What psychophysical characteristics should have?
18. What is the significance of the authenticity of sensations for you and overcoming your own psychophysical barriers in a long-distance winter mountain run?
19. Do you think the experience of participating in ultramarathon affects the social identity of ultramarathoners?
20. What does exposure to your body to a severe borderline situation mean to you? Where are these limits in your case?
21. How important is running in your life in relation to other spheres (e.g., work, family, religion, politics etc.)?
22. Do you exchange experiences with other athletes during the ultramarathon? If yes, what does this mean to you?

**Equipment and safety:**

*Mandatory equipment:*

Each participant, during the whole run, must carry the mandatory equipment comprising:

– clothing adapted to winter weather conditions
– watertight and windproof long sleeve jacket
– one pair of gloves (watertight, windproof)
– hat/buff
– additional warming long sleeve layer
– backpack or waist bag containing all the mandatory equipment
– headlamp with fully charged batteries
– pair of chemical warmers (packed)
– water bottle with at least 1 L capacity
– switched on and charged mobile phone (with roaming) containing emergency and organizer’s numbers
– ID or valid passport
– space blanket with a minimum size of 140 × 200 cm
– whistle
– red flashing light (attached to a backpack, waist bag, or back clothing item)
– starting number (attached in a visible place—provided by the organizer)
– route map—provided by the organizer

Lack of any item from a mandatory equipment list during the competition (from start line to finish line) may result in disqualification or time penalties.

*Recommended equipment:*

– mini crampons (highly recommended)
– snowshoes
– GPS receiver with a route map
– crampons
– footwear with spikes
– footwear with Gore-tex membrane
– gaiters
– waterproof and windproof pants
– trekking poles

*Safety*

1. The competition is safeguarded by the Karkonosze Mountain Volunteer Ambulance Service rescuers.
2. The Karkonosze Mountain Volunteer Ambulance Service emergency vehicle with visual or audible warning signals is given absolute priority regardless the direction of travel.
3. Emergency foot patrol with means of transport is given absolute priority on the route.
4. For safety reasons, the organizer may change or shorten the route or postpone the competition until the back-up day. The route may be shortened by the organizers together with GOPR rescuers on the day prior to or during the competition.

In the case the run is interrupted, the classification will be based on the so called split times (measured at refreshment stations) or last times that were recorded by Officials during the run.

5. In the case of extremely unfavorable atmospheric conditions the competition may be cancelled. The run may be cancelled by the organizer in consultation with the Karkonosze GOPR rescuers.

If the run is cancelled for reasons beyond the organizer’s control, the participation fee will not be reimbursed.

6. In the case a participant exceeds the time limit set for each refreshment and Officials station, he or she will not be allowed to continue with the race.
7. Participants withdrawing from the race will have a chance to stay in a shelter. They will be allowed to leave a shelter only under the supervision of a person designated by the organizer. Participants will reach Karpacz city in small groups. Persons suffering from excessive loss of temperature or those who have lost their strength will be ensured overnight accommodation in a shelter.

A participant will be allowed to leave a shelter on their own upon submitting a written resignation from the competition and declaration confirming intent to leave a shelter at their own risk.

8. During the run, participants must pay particular attention to other runners. Weather conditions may be very difficult. A sudden loss of temperature, fog, and snowfall may put the well-being of participants at risk. We kindly ask participants to ensure mutual assistance. If the need arises, please inform the organizer, Officials, or GOPR rescuers staying on the route and in the designated stations, about the problem.
9. A team consisting of at least two Officials designated by the organizer will follow the last competitor.
10. A route map provided for each participant will include emergency and organizer’s numbers.
11. Participants will be informed about any icy areas occurring on the route. These places will be clearly marked and guarded by additional Officials.
12. Each participant must sign a declaration confirming participation in the run at their own responsibility and risk.
13. Participants must use district and national roads in a way indicated or imposed by the organizer, Police, or Municipal Guard. They are also obliged to comply with instructions of the organizer, Police, or Municipal Guard.
14. Participants undertake to be extremely careful when passing asphalt roads and railways and running through asphalt roads as well as to fully comply with warning signs of the organizer and instructions of the Police and the organizer’s security personnel. When running through public roads participants undertake to abide by the Traffic Code.
